# Insecticide resistance in indoor and outdoor-resting *Anopheles gambiae* in Northern Ghana

**DOI:** 10.1186/s12936-020-03388-1

**Published:** 2020-08-31

**Authors:** Majidah Hamid-Adiamoh, Alfred Amambua-Ngwa, Davis Nwakanma, Umberto D’Alessandro, Gordon A. Awandare, Yaw A. Afrane

**Affiliations:** 1 West African Centre for Cell Biology of Infectious Pathogens (WACCBIP) and Department of Biochemistry, Cell and Molecular, University of Ghana, Legon, Ghana; 2 Medical Research Council Unit, The Gambia at the London School of Hygiene & Tropical Medicine, Banjul, Gambia; 3 Department of Medical Microbiology, College of Health Sciences, University of Ghana, Legon, Accra, Ghana

**Keywords:** *Anopheles gambiae*, Insecticide resistance, Indoor and outdoor behavior, Northern Ghana

## Abstract

**Background:**

Selection pressure from continued exposure to insecticides drives development of insecticide resistance and changes in resting behaviour of malaria vectors. There is need to understand how resistance drives changes in resting behaviour within vector species. The association between insecticide resistance and resting behaviour of *Anopheles gambiae* sensu lato (*s.l.*) in Northern Ghana was examined.

**Methods:**

F_1_ progenies from adult mosquitoes collected indoors and outdoors were exposed to DDT, deltamethrin, malathion and bendiocarb using WHO insecticide susceptibility tests. Insecticide resistance markers including voltage-gated sodium channel (Vgsc)-*1014F*, *Vgsc*-*1014S*, *Vgsc*-*1575Y*, glutathione-*S*-transferase epsilon 2 (*GSTe2*)-*114T* and acetylcholinesterase (*Ace1*)-*119S*, as well as blood meal sources were investigated using PCR methods. Activities of metabolic enzymes, acetylcholine esterase (AChE), non-specific β-esterases, glutathione-*S*-transferase (GST) and monooxygenases were measured from unexposed F_1_ progenies using microplate assays.

**Results:**

Susceptibility of *Anopheles coluzzii* to deltamethrin 24 h post-exposure was significantly higher in indoor (mortality = 5%) than outdoor (mortality = 2.5%) populations (P = 0.02). Mosquitoes were fully susceptible to malathion (mortality: indoor = 98%, outdoor = 100%). Susceptibility to DDT was significantly higher in outdoor (mortality = 9%) than indoor (mortality = 0%) mosquitoes (P = 0.006). Mosquitoes were also found with suspected resistance to bendiocarb but mortality was not statistically different (mortality: indoor = 90%, outdoor = 95%. P = 0.30). Frequencies of all resistance alleles were higher in F_1_ outdoor (0.11–0.85) than indoor (0.04–0.65) mosquito populations, while *Vgsc*-*1014F* in F_0_ *An. gambiae* sensu stricto (*s.s*) was significantly associated with outdoor-resting behaviour (P = 0.01). Activities of non-specific β-esterase enzymes were significantly higher in outdoor than indoor mosquitoes (Mean enzyme activity: Outdoor = : 1.70/mg protein; Indoor = 1.35/mg protein. P < 0.0001). AChE activity was also more elevated in outdoor (0.62/mg protein) than indoor (0.57/mg protein) mosquitoes but this was not significant (P = 0.08). Human blood index (HBI) was predominantly detected in indoor (18%) than outdoor mosquito populations (3%).

**Conclusions:**

The overall results did not establish that there was a significant preference of resistant malaria vectors to solely rest indoors or outdoors, but varied depending on the resistant alleles present. Phenotypic resistance was higher in indoor than outdoor-resting mosquitoes, but genotypic and metabolic resistance levels were higher in outdoor than the indoor populations. Continued monitoring of changes in resting behaviour within *An. gambiae s.l.* populations is recommended.

## Background

Malaria control and elimination efforts rely heavily on vector control interventions, more specifically on long-lasting insecticidal nets (LLINs) and indoor residual spraying (IRS), that involve the use of insecticides [1]. The scale-up of LLIN, has contributed significantly to the decline of malaria burden observed over the last 10–15 years in sub-Saharan Africa [2]. Unfortunately, malaria vectors have developed resistance to the insecticides employed in vector control programmes and indeed to almost all the classes of available insecticides [3]. Insecticide use has been associated with widespread physiological resistance and behavioural changes of malaria vectors which may contribute in maintaining residual malaria transmission [4, 5]. IRS and LLINs are meant to provoke a knock down or mortal effect on vectors upon contact, targeting their classical anthropophilic (human feeding), late night indoor biting (endophagic) and indoor resting (endophilic) behaviours [6, 7]. This applies specifically to the most efficient malaria vectors, namely *Anopheles arabiensis*, *Anopheles coluzzii*, *Anopheles gambiae* sensu stricto (*s.s.*) and *Anopheles funestus*. Contrary to expectations, in settings where IRS and LLINs were extensively deployed, highly anthropophilic, late-indoor biting and indoor resting vectors have switched to animal feeding and outdoor human feeding following the deployment of vector control activities [8, 9]. For instance, *An. gambiae* sensu lato (*s.l.*) populations in Bioko Island [10], Ghana [11], Senegal [12] and Tanzania [8] increased outdoor feeding behaviour following extensive intervention with IRS and LLINs. Outdoor biting was also found in naturally endophilic *An. funestus* populations in Western Kenya [13]. Furthermore, vector populations have adapted to early and early-morning biting, targeting a time when humans are not protected by LLINs [14].

Intriguingly, recent studies done in areas of high IRS and LLINs coverage have shown concurrent indoor and outdoor feeding behaviour within sibling species of *An. gambiae s.l.* from Benin [15], Ethiopia [16], Libreville [17], Tanzania [8] and Western Kenya [13]. However, there is little evidence of intra-species consistency or differences in insecticide-driven vector resting behaviour. It is plausible that insecticide pressure may select for behavioural changes within species, such that resistant mosquitoes feed and survive indoors while susceptible mosquitoes adopt exophilic behaviour. This can be further modulated by variation in molecular mechanisms that enable survival against insecticides.

Target site and metabolic resistance mechanisms have been shown to confer resistance to insecticides in *An. gambiae s.l.* [18–20]. Target site resistance involves mutation in the voltage-gated sodium channel (Vgsc) gene, mediating resistance to dichlorodiphenyltrichloroethane (DDT) and pyrethroids [21, 22], as well as acetylcholinesterase (ACE), responsible for carbamate and organophosphate resistance [23, 24]. Increased detoxifying activities of metabolic enzyme families including non-specific esterases, glutathione-*S*-transferases (GSTs) and monooxygenases (cytochrome P450s) were associated with resistance to the various malaria control insecticides [25, 26]. Several markers have been identified and widely used for resistance surveillance. In *An. gambiae s.l.*, knockdown resistance (kdr) *Vgsc*-*1014F*, *Vgsc*-*1014S*, *Vgsc*-*1575Y* and glutathione-s-transferase epsilon 2 (*GSTe2)*-*114T* are markers associated with DDT and pyrethroid resistance [27, 28], whereas *Ace1*-*119S* is linked to organophosphates and carbamates resistance [29]. The prevalence of the resistance phenotypes and polymorphisms, as well as enzymatic activities, in association with vector behavioural patterns may help understand the effect of vector interventions and strategies to improve efficacy in specific malaria endemic populations.

Malaria transmission is spatio-temporally heterogenous in Ghana with intensities highly driven along different ecological zones [30, 31]. Recently, malaria prevalence in under 5 years was estimated to be about 40% [32]. Vector control with IRS and LLINs has been a key strategy for malaria control, where an estimated LLIN usage of 73% was previously recorded and reduction of malaria burden has been attributed to the effectiveness of these tools [32–34]. *Anopheles coluzzii*, *An. gambiae* s.s and *An*. *funestus* are the main vector species responsible for transmission [35, 36]. DDT, pyrethroid and carbamate resistance have been reported in these vectors across the country [11, 37], but vectors remain susceptible to pirimiphos-methyl, an organophosphate [38].

Northern Ghana is a hyperendemic transmission setting where entomological inoculation rate (EIR) of > 150 infective bites/person/year has been documented [36]. Currently, IRS and LLINs are extensively being deployed annually in Northern Ghana but the impact of these measures on the behaviour and insecticide resistance in vector populations remains unclear. This study therefore investigated the association between resting behaviour of members of *An. gambiae s.l*. and insecticide resistance and its contribution to residual malaria transmission in Northern Ghana.

## Methods

### Study sites

**Fig. 1:**
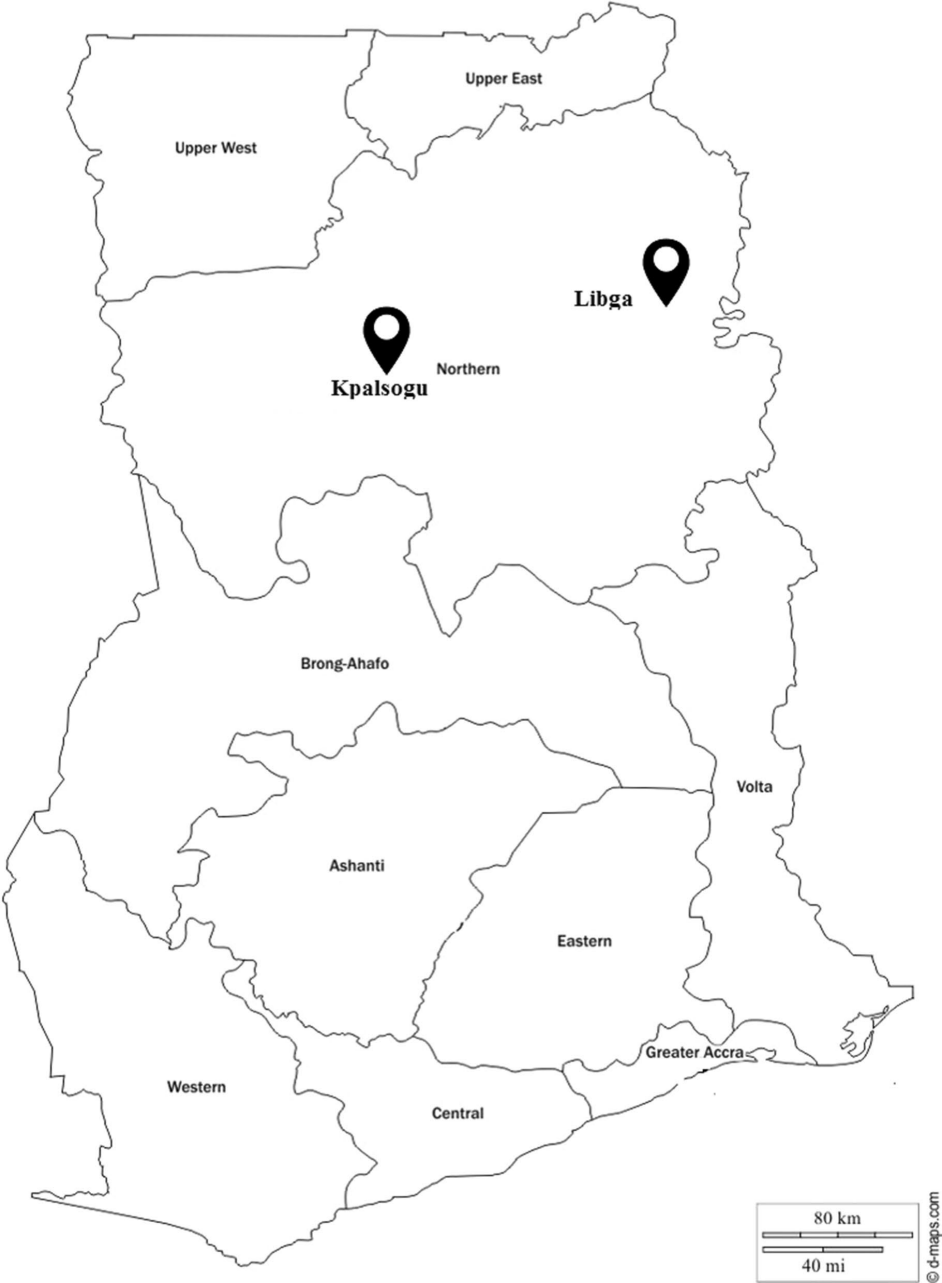
Map of study sites

The study was conducted in two rural communities in Northern Ghana, which are 16 km apart, Kpalsogu (9.33° N, 1.02° W) and Libga (9.35° N, 0.51° W) (Fig. 1). Northern Ghana was chosen because the region continues to experience a high malaria burden, with incidence rate of about 40% in under-five children [39] and persistent high EIR of > 150 infective bites/person/year [36, 40], despite scaled-up malaria control interventions. Kpasolgu is one of the sites for annual IRS conducted by the President Malaria Initiative (PMI) and Ghana National Malaria Control Programme (NMCP) since 2008. However, IRS started in Libga in 2008 but was discontinued from 2014. Both communities are in close proximity to dams linked to an irrigation scheme which allows uninterrupted farming activities throughout the year but also supports perennial breeding of mosquitoes [36]. Malaria transmission is seasonal in the areas without irrigation [41].

### Mosquito collections and rearing in the insectary

Indoor and outdoor mosquito collections were conducted in July–November 2017 from each site every other day. Collections were done between 06:00 h and 09:00 h. Live indoor-resting mosquitoes were sampled using prokopack electrical aspirators [42]. Pit traps [43] were constructed outside houses to attract live outdoor-resting mosquitoes which were later collected with prokopack aspirators. Four pit traps were constructed in each village. Each trap was placed about 5 m from each compound and the houses were 50 m apart from each other. Both indoor and outdoor collections were done in 6–8 randomly selected compounds in each community. Mosquitoes were transferred into paper cups labeled as per their resting locations.

Mosquitoes were immediately transported to the insectary for morphological identification of species and abdominal status using taxonomic keys [44]. All blood fed, half-gravid and gravid F_0_ female *An. gambiae s.l.* were kept in cages to lay eggs. They were provided with laying pads, made of filter paper on top of a wet cotton wool in a Petri dish. Eggs were subsequently allowed to hatch and larvae reared to adult stage.

### Insecticide susceptibility bioassay

Batches of 20–25 emerging F_1_ adult females (2–5 days old) from 480 wild-caught F_0_ females, were exposed to insecticide-impregnated papers containing 0.05% deltamethrin, 5% malathion, 0.1% bendiocarb and 4% DDT following standard World Health Organization (WHO) tube test protocol [45]. Two batches of the same number of mosquitoes were exposed to untreated test papers as negative controls. Mosquitoes were then supplied with 10% sugar solution in a holding tube and mortality after 24 h was recorded and scored according to WHO protocol [45]. Dead and surviving mosquitoes were separately stored in 1.5 ml Eppendorf tubes with silica gel for subsequent molecular tests for insecticide resistance mechanisms.

### Anopheles species identification

Genotypic DNA was extracted from the legs of individual F_0_ and F_1_ female mosquitoes using Qiagen QIAxtractor robot. Species Identification to the molecular level was carried out as previously done [46, 47]. All phenotyped F_1_ and F_0_ *An. gambiae s.l.* mosquitoes were analysed for species identification. Primers included in the reaction were those that detect sibling species of *An. gambiae* complex, including *An. arabiensis*, *An. coluzzii*. *An. gambiae s.s* and *Anopheles melas*; which are the relevant vectors of malaria in Ghana [48].

### Analyses of target site modifications

From each resting location, 50 mosquitoes were selected per insecticide for genotyping of insecticide resistance polymorphisms in phenotyped mosquitoes. Selection was done using dplyr package in R (cran.r-project.org). Similarly, all 480 F_0_ that lay eggs and the remaining wild mosquitoes that were not selected for egg laying were processed for genotypic assessment of insecticide resistance mechanisms. Single nucleotide polymorphism (SNP) markers of insecticide resistance were screened from DNA of each specimen using a TaqMan SNP genotyping probe-based assays [49]. These markers include *Vgsc*-*1014F*, *Vgsc*-*1014S* and *Vgsc*-*1575Y* for target site resistance mutations to DDT and pyrethroids in voltage-gated sodium channel [21, 22, 27]; *Ace1*-*119S* mutation, marker of resistance to carbamates and organophosphates [29] and *Gste2*-*114T*, a molecular marker of metabolic resistance to DDT [28]. Analysis of allele frequencies of *kdr* mutations was conducted in the F1 generation of the *An. coluzzii* alone because they were the majority species encountered in the study sites.

### Metabolic enzyme activity assays

Other subsets of emerging F_1_ adult females (2–5 days old) were immediately frozen in − 20 °C for biochemical assays. The frozen specimens were analysed for activities of metabolic enzymes including AChE, non-specific β-esterases, GSTs and monooxygenases (oxidases). 50 F_1_ mosquitoes were analysed from each of the study localities and they were not exposed to any insecticide prior to the assays. Microplate assay standard protocols as described [50] were followed for each enzyme, where all assays were run in triplicates and along with Kisumu strain as susceptible control population.

Briefly, individual whole adult mosquitoes (enzyme source) were homogenized in potassium phosphate (KPO_4_) buffer and substrates to respective enzymes were added as well as chromogenic agents. Absorbance was measured using Varioskan Lux multimode microplate reader (Thermo Scientific) at specific wavelengths depending on the enzyme being measured. Acetylcholine esterase was measured at 414 nm in the presence of acetylthiocholine iodide (ATCH) as substrate; while β-esterases at 540 nm in the presence of β-naphthyl acetate. Monooxygenes (cytochrome P450) level was determined using 3, 3′, 5,5′-Tetramethyl-Benzidine Dihydrochloride (TMBZ) and absorbance captured at 620 nm. Lastly, glutathione-*S*-transferase with 1-chloro-2, 4′-dinitrobenzene (cDNB) at 340 nm. Total protein from individual mosquitoes was also analysed to standardize the mean enzyme activity of the test samples.

### Analysis of blood meal sources

Blood meal origins were determined from DNA extracted from the abdomens of blood-fed F_0_ mosquitoes using the multiplex PCR protocol [51] modified by including primers that could amplify donkey and horse. This assay involves amplification of mitochondrial cytochrome B of *An. gambiae* vertebrate hosts including cow, dog, donkey, goat, human, horse and pig from a single mosquito specimen.

### Data analysis

Data from both study sites were pooled together as there was no significant difference in the results obtained. The level of insecticide susceptibility of mosquitoes was evaluated following WHO 2016 criteria [45]. Pearson’s Chi squared test was used to determine the differences in mortality to insecticides by resistance allele and their frequencies between indoor and outdoor mosquito populations. Odds ratio was applied to determine the association between resistance phenotype and frequency of resistance alleles in F_1_ mosquito populations exposed to insecticides.

Mean activities of each enzyme per mg of protein were compared between mosquitoes from the two resting locations and the reference susceptible strain, using one-way analysis of variance (ANOVA) with Holm–Sidak’s multiple comparisons test. The mean enzyme activities between indoor and outdoor mosquitoes were compared using Mann–Whitney test. Human (HBI) and animal (BBI) blood indices were each calculated as total number of mosquitoes positive for human and animal DNA as a proportion of all blood fed mosquitoes expressed in percentage. All statistical analyses were performed using Stata/IC 15.0 (2017 StataCorp LP) and GraphPad Prism 8.0.1 software. P value of < 0.05 was considered significant in all data interpretations.

## Results

### Anopheles mosquito species composition

A total of 3675 mosquitoes were collected during the study. Of these, 1122 (31%) were female *An. gambiae s.l.*, 2358 (64%) *An. funestus* complex and 195 (5%) Culicine mosquitoes. The majority (58%, 652) of *An. gambiae s.l*. mosquitoes were found resting outdoors than indoors (42%, 470). *Anopheles coluzzii* was the predominant species, both indoors (36%, 375) and outdoors (39%, 413), followed by *An. arabiensis* (3%, 33) indoors and outdoors (12%, 125), and *An. gambiae s.s.* indoors (2%, 27) and outdoors (8%, 83). Five (5) hybrids of *An. coluzzii/gambiae s.s.* were also identified.

### Phenotypic resistance in F_1_*Anopheles coluzzii* populations

**Fig. 2:**
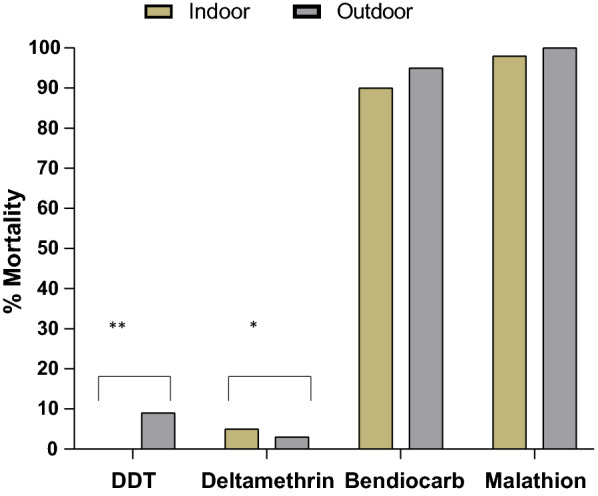
Phenotypic resistance to the four insecticides tested in indoor and outdoor mosquitoes. *P = 0.02. **P = 0.006

Overall, a total of 780 mosquitoes (indoor: 380, outdoor: 400) were exposed to insecticides (Additional file 1: Table S1) from about 160 (indoor) and 320 (outdoor) F_0_ adults that successfully laid eggs. Species identification of all phenotyped samples revealed 98% of F_1_ progeny were *An. coluzzii* both indoors and outdoors while the remaining species (*An. arabiensis* and *An. gambiae*) represented 2%. Mortality was generally higher in outdoor mosquitoes than the indoor populations. A 24-h post-exposure mortality of 0% and 9% (95% CI 3–12%) was observed for DDT with progeny of mosquitoes from indoor and outdoor respectively (Fig. 2) and this difference was statistically significant (Pearson *X*^2^ = 7.58, df = 1, P = 0.006). Progeny of mosquitoes exposed to deltamethrin showed an overall mortality of 5% (95% CI 1–12%) for indoor mosquitoes and 2.5% (95% CI 8–34%) for outdoor-resting mosquitoes (Pearson *X*^2^ = 5.44, df = 1, P = 0.02).

The indoor and outdoor mosquitoes exposed to bendiocarb showed suspected resistance with mortality of 90% (95% CI 64–95%) in the indoor population and 95% (95% CI 87–100%) in the outdoor population (Pearson *X*^2^ = 1.07, df = 1, P = 0.30). Both the indoor and outdoor populations were fully susceptible to malathion, with 98% and 100% (95% CI 87–100%) mortality for indoor and outdoor mosquitoes, respectively (Pearson *X*^2^ = 2.02, df = 1, P = 0.16). There was no observed mortality (0%) in the controls for all insecticides tested.

### Detection of resistance alleles in F_1_*An. coluzzii* populations

Resistance-associated allele frequencies were higher in outdoor-resting mosquitoes than the indoor population (Table 1). *Vgsc*-*1014F* and *GSTe2*-*114T* alleles were the most common in both phenotypically resistant and susceptible indoor and outdoor mosquitoes. In the deltamethrin-resistant mosquitoes, *Vgsc*-*1014F* frequency was 0.65 (indoor) and 0.67 (outdoor). However in the DDT-resistant mosquitoes, *Vgsc*-*1014F* frequency was 0.65 (indoor) and 0.73 (outdoor). These observed differences were not statistically significant between the indoor and outdoor mosquito populations (Deltamethrin: Pearson *X*^2^ = 0.22, df = 1, P = 0.64. DDT: Pearson *X*^2^ = 0.41, df = 1, P = 0.52). The carriage of *Vgsc*-*1014F* mutation was strongly associated with resistance to deltamethrin (OR = 5.46, P = 0.001, 95% CI 1.94–15.41) but not with DDT resistance (OR = 0.69, P = 0.75, 95% CI 0.066–7.14). No *Vgsc*-*1014S* allele was detected in any of the mosquitoes.

**Table 1:** Frequencies (proportions) of resistance alleles in indoor and outdoor F_1_ *An. coluzzii* populations based on insecticide resistance phenotypes (dead and alive)

Deltamethrin	Vgsc-1014F		Vgsc-1575Y		GSTe2-114T		Ace1-119S	
	Dead	Alive	Dead	Alive	Dead	Alive	Dead	Alive
	(N = 8)	(N = 81)	(N = 8)	(N = 82)	(N = 8)	(N = 82)		
Indoor	0.5	0.65	0	0.07	0.5	0.62		
Outdoor	1	0.67	1	0.27	1	0.84		

DDT	(N = 4)	(N = 49)	(N = 0)	(N = 50)	(N = 4)	(N = 52)		
Indoor	0	0.65	0	0.04	0	0.56		
Outdoor	0.75	0.73	0	0.11	1	0.85		

Bendiocarb							(N = 55)	(N = 3)
Indoor							0.06	1
Outdoor							0	1

Malathion							(N = 59)	(N = 1)
Indoor							0.08	0
Outdoor							0.12	0

N represents overall number of mosquito population positive by PCR for individual resistance allele in each indoor and outdoor populations

*Vgsc*-*1575Y* mutation was detected mainly in the deltamethrin-resistant outdoor *An. coluzzii* populations (frequency = 0.27). *GSTe2*-*114T* mutation was significantly higher in outdoor-resting (0.85) mosquitoes than the indoor (0.56) DDT-resistant mosquitoes (Pearson *X*^*2*^ = 5.73, df = 1, P = 0.02). This mutation was also identified in mosquitoes resistant to deltamethrin (indoor = 0.62, outdoor = 0.84).

*Ace1*-*119S* was detected in a single indoor and an outdoor *An. coluzzii* specimens that survived bendiocarb exposure. It was also found in a single bendiocarb-resistant outdoor mosquito. The allele was detected only in malathion-susceptible mosquitoes at frequency of 0.08 (indoor) and 0.12(outdoor) with no significant difference (Pearson *X*^2^ = 0.003, df = 1, P = 0.96).

### Detection of resistance alleles in F_0_*An. gambiae* populations

The frequency of resistance alleles between the indoor and outdoor mosquitoes varied by mosquito species. Whereas *Vgsc*-*1014S* was not detected in the F_1_ *An. coluzzii*, it was observed mainly in the F_0_ *An. arabiensis* resting outdoors. *Vgsc*-*1014F* mutation was significantly higher in outdoor (0.99) resting mosquitoes compared to those indoors (0.77) in *An. gambiae s.s.* (Pearson *X*^*2*^= 31.6, df = 2, P = 0.001) (Table 2). There was an indication of association of outdoor-resting behaviour with resistance in *An. gambiae s.s*. population carrying the *Vgsc*-*1014F* mutation (OR = 0.05, P = 0.01, 95% CI 0.005–0.419). Although, *An. coluzzii* was the predominant species collected both indoors and outdoors, the difference in the frequency of this mutation in indoor (0.65) and outdoor (0.70) populations was not statistically significant (Pearson *X*^*2*^= 0.7, df = 2, P = 0.4). However, the higher prevalence of the mutation in indoor (0.48) than the outdoor (0.21) *An. arabiensis* population was significant (Pearson *X*^2^ = 6.42, df = 2, P = 0.04). *Vgsc*-*1014S* was mainly found in indoor *An. arabiensis* (0.42).

**Table 2:** Frequencies (proportions) of resistance alleles in the wild F_0_ indoor and outdoor *An. gambiae* sl populations

	Vgsc-1014F		*Vgsc*-*1014S*		*Vgsc*-*1575Y*		*GSTe2*-*114T*		*Ace1*-*119S*	
	Indoor	Outdoor	Indoor	Outdoor	Indoor	Outdoor	Indoor	Outdoor	Indoor	Outdoor
*An. arabiensis*	0.48	0.21	0.42	0.39	0	0.02	0.13	0.07	0	0.01
	N = 21	N = 75	N = 19	N = 97		N = 119	N = 15	N = 54		N = 26
*An. coluzzii*	0.65	0.7	0.01	0.01	0.21	0.2	0.84	0.86	0.01	0.01
	N = 352	N = 401	N = 125	N = 122	N = 358	N = 403	N = 213	N = 307	N = 364	N = 401
*An. gambiae s.s*	0.77	0.99	0	0.5	0.3	0.18	0.25	0.13	0.25	0.31
	N = 22	N = 74	N = 5	N = 2	N = 23	N = 76	N = 12	N = 56	N = 24	N = 80
*An. coluzzii/gambiae s.s*	1	1								
	N = 3	N = 2								

*Vgsc*-*1575Y* was detected at an almost similar level (frequencies: Indoor = 0.21, outdoor = 0.2) in *An. coluzii* populations. Further, no significant difference was observed in indoor (0.30) and outdoor (0.18) *An. gambiae s.s*. (Pearson *X*^*2*^= 1.2, df = 1, P = 0.27).

*Ace1*-*119S* mutation was most frequent in *An. gambiae s.s.*, although there was no statistically significant difference in the frequencies between indoor (0.25) and outdoor (0.31) populations (Pearson *X*^*2*^= 0.2, df = 1, P = 0. 65). The prevalence was 0.1 in indoor and outdoor *An. coluzzii*.

### Metabolic enzyme activities in F_1_*An. coluzzii* populations

**Fig. 3:**
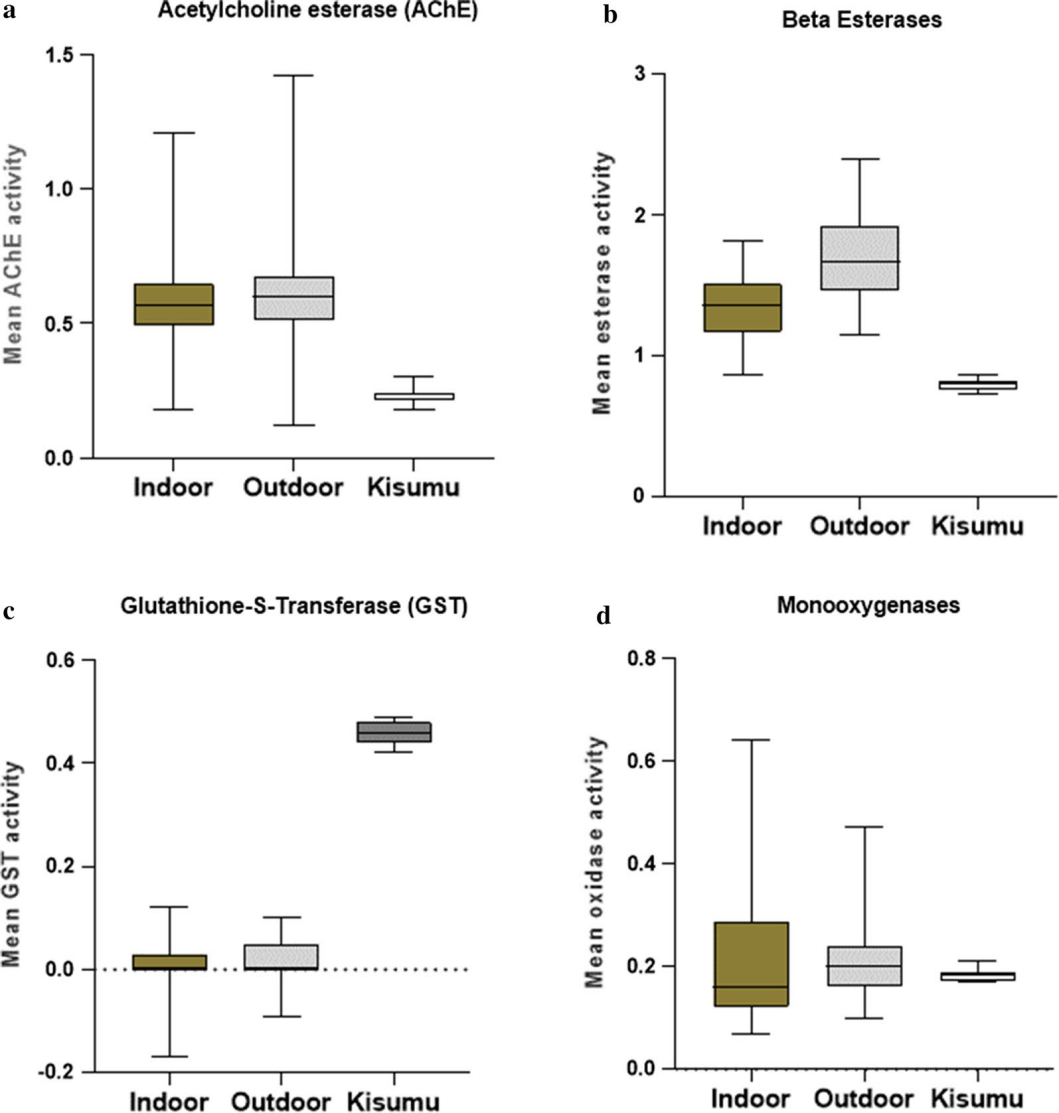
Mean enzyme activities observed in individual enzymes in indoor and outdoor mosquitoes and the susceptible reference strain, Kisumu

An overall highly significant elevated levels of AChE (F_2, 237_ = 55.93, P < 0.0001) and β-esterase (F_2, 237_ = 159.0, P < 0.0001) activities were observed in both indoor and outdoor mosquito populations compared to the susceptible reference strain, Kisumu (Figs. 3a–d). Conversely, in both mosquito populations, the activities of the GSTs and monooxygenases were less relative to Kisumu but this was not significant in monooxygenase activity (F_2, 237_ = 0.6589, P = 0.52).

AChE activity was not significantly higher in the outdoor (0.62/mg protein) than the indoor (0.57/mg protein) population (Mann–Whitney U = 5037, Z = − 1.73, P = 0.08). The elevation in enzyme activity was found (Table 3) to be 2.48-fold (indoor) and 2.7 fold (outdoor) significantly higher than in Kisumu (P < 0.0001). Similarly, non-specific β-esterase activity in the outdoor-resting mosquitoes (1.70/mg protein) was significantly more than the indoor mosquitoes (1.35) (Mann–Whitney U = 0.5, Z = − 8.33, P < 0.0001); with 1.69 (indoor) and 2.13 (outdoor) significant fold changes (P < 0.0001). No significant difference was detected in the level of GST activity between the two mosquito populations (indoor: 0.01/mg protein, outdoor: 0.02/mg protein, (Mann–Whitney U = 5709, Z = − 0.29, P = 0.78). Monooxygenase activities also showed a similar level in both indoor and outdoor mosquitoes (mean activity = 0.21/mg protein, (Mann–Whitney U = 4989, Z = − 1.84, P = 0.07). The fold difference in monooxygenase activities in Kisumu population (1.11) was not statistically significant (P = 0.59).

**Table 3:** Mean activities of individual enzyme and the fold change in mosquito populations relative to Kisumu

Enzyme	Mosquito population	Mean enzyme activity (95% CI)	Fold change	P-value
AChE	Kisumu	0.23 (0.22–0.24)		
	Indoor	0.57 (0.54-–0.60)	2.48	< 0.0001
	Outdoor	0.62 (0.58–0.66)	2.7	< 0.0001
β-esterase	Kisumu	0.80(0.78–0.81)		
	Indoor	1.35 (1.31–1.39)	1.69	< 0.0001
	Outdoor	1.70 (1.65–1.76)	2.13	< 0.0001
GST	Kisumu	0.46 (0.45–0.47)		
	Indoor	0.01 (0.0–0.01)	0.02	< 0.0001
	Outdoor	0.02 (0.01–0.02)	0.04	< 0.0001
Monooxygenase	Kisumu	0.19 (0.18–0.19)		
	Indoor	0.21 (0.18–0.23)	1.11	0.59
	Outdoor	0.21 (0.19–0.22)	1.11	0.59

### Host blood meal sources of wild F_0_ mosquitoes

A total of 165 out of 214 blood-fed mosquitoes were successfully identified to have fed either on human or animal hosts. The overall vertebrate positivity rate was higher in indoor-resting mosquitoes (Table 4), predominantly in *An. coluzzii*, which was the most abundant species in the study sites. Overall human blood index (HBI) was 21% and again more prominent in indoor (18%) than outdoor (3%) mosquitoes. 69% of HBI was detected from indoor *An. coluzzii*, followed by 17% in outdoor *An. coluzzii*. *Anopheles arabiensis* was found with only 9% (indoor) and 1% (outdoor) HBI, while 1% HBI was identified in indoor *An. gambiae* only.

**Table 4:** Proportion of blood meal origin of the indoor and outdoor-resting mosquito populations

	*An. arabiensis*	*An. coluzzii*	*An. gambiae*
	Proportion (n)	Proportion (n)	Proportion (n)
Human			
Indoor	0.02 (3)	0.15 (24)	0.01 (1)
Outdoor	0.01 (1)	6 (0.04)	0
Cow			
Indoor	0	0.01 (2)	0
Outdoor	0	0.01 (2)	0
Dog			
Indoor	0	0.05 (8)	0
Outdoor	0	0.01 (2)	0
Donkey			
Indoor	0	0.04 (7)	0
Outdoor	0	0.01 (1)	0
Goat			
Indoor	0.04 (7)	0.36 (59)	0.01 (2)
Outdoor	0.07 (12)	0.08 (13)	0.01 (1)
Horse			
Indoor	0	0.02 (3)	0
Outdoor	0.01 (2)	0	0
Pig			
Indoor	0	0.01 (1)	0
Outdoor	0.01 (2)	0.01 (1)	0
Mixed hosts			
Indoor (human + goat)	0	0.01 (1)	0
Outdoor (cow + goat)	0	0.02 (3)	0.01 (1)

The principal animal blood meal source was from goat, representing 36% of indoor against 8% of outdoor-resting *An. coluzzii*. The other animal blood sources included cows, dogs, donkeys, horses and pigs and were mainly detected in indoor *An. coluzzii* specimens. Fewer (4%) *An. arabiensis* resting indoors fed on animal blood compared to the outdoor population (10%), similar to *An. gambiae s.s.* indoor (0.6%) and outdoor (1.2%) population. Mixed human and goat blood meal was identified from a single indoor *An. coluzzii* specimen. Also, mixed cow and goat meal were found in three outdoor *An. coluzzii* specimens and one outdoor *An. gambiae s.s.* specimen.

## Discussion

In this study, phenotypic resistance was found to be higher in indoor-resting than outdoor-resting *An. coluzzii* progeny. Higher resistance-associated alleles and elevated activities of two metabolic enzymes were however recorded in the outdoor-resting population. Overall, there was not a significant association between insecticide resistance and resting location of the mosquito populations; but there was a tendency for *An. coluzzii* to rest indoors when phenotypically resistant to DDT and *An. gambiae s.s.* to rest outdoors when resistance was genotypically due to *Vgsc*-*1014F* mutation. Enhanced AChE and β-esterases activities were also prominent in outdoor-resting F_1_ *An. coluzzii*. Moreover, human and animal blood meal indices were higher in indoor than the outdoor mosquito population but with no statistical significance.

A probe into insecticide-driven genetic adaptation in vector population at intra-species level was a main interest in this study. It was hypothesized that higher resistance levels in indoor compared to the outdoor populations, due to increased contact with insecticide which amplifies their propensity to develop resistance [52]. This was mainly evident in the F_1_ *An. coluzzii* populations exposed to insecticides where indoor mosquitoes were less susceptible to three out of four insecticides. Phenotypic resistance was especially high in DDT-exposed indoor populations while genotypic resistance and enzymatic activities were more prevalent in all outdoor F_1_ mosquito populations. This may be due to selection pressure from prolonged use of DDT for IRS until it was recently switched to pirimiphos-methyl, an organophosphate. Cross-resistance from other pyrethroids used in LLINs may also contribute [53].

This study showed an association between outdoor-resting behaviour in F_0_ An*. gambiae s.s.* population and *Vgsc*-*1014F* mutation. High frequencies of resistance markers associated with DDT resistance, *Vgsc*-*1575Y* and *GSTe2*-*114T*, were also observed in both F_0_ and F1 indoor and outdoor *An. coluzzii* populations. These may result from selection pressure due to the widespread use of similar insecticides for both public health and agriculture. As noted, year-round agriculture is practiced in the study sites where crops as rice and tomatoes are specifically cultivated with pesticides such as pyrethroids and carbamates predominantly used for pest control [54, 55]. This could further explain why there were no significant difference in frequencies between the indoor and outdoor populations. Notably, resistance has been previously reported at varying levels to DDT and deltamethrin across all vector species in Ghana [53, 56] and the neighboring countries including Benin [19], Burkina Faso [27] and Togo [57]. High frequencies of resistance loci may compromise the effectiveness of vector control in the study areas that could subsequently accentuate residual transmission [41].

Target site polymorphisms may not fully explain resistance in vector populations [58], thus the possible metabolic mechanisms involved were probed. Significant increase in the activities of AChE and β-esterases were identified, both of which have been associated with resistance to the insecticides tested [59]. Consistently, the observed fold change in β-esterases activities which was significantly higher than the susceptible strain, may demonstrate a possible role in deltamethrin resistance in the vector populations as previously reported [60, 61]. On the other hand, the decreased levels of GSTs and monooxygenases detected may indicate that they do not contribute to the DDT and deltamethrin resistance in the study mosquito populations. Therefore, genotypic mechanism alone may be mediating the documented DDT resistance in these mosquito populations. Interestingly, an increased activity of AChE was identified despite low level of *Ace1*-*119S* and no phenotypic resistance in the carbamate and organophosphate insecticides in the study. This may likely reflect other role of this enzyme, which may not be related to resistance in the vector population. Since there was no documented use of carbamate insecticide for IRS in this region except for agricultural use [30], perhaps this resistance selection may be from agriculture use.

Human and animal blood indices were found to be higher in indoor-resting mosquitoes than the outdoor population despite a higher outdoor collection. This indicates that in spite of the fact that the study areas were under high IRS and LLINs interventions, mosquitoes were able to have either fed on their host indoors or outdoors and still successfully rested indoors despite interventions; thus retained their indoor-resting behaviour. Plausibly, the blood-fed endophilic population could be among the indoor-resistant populations that are capable of maintaining contact with insecticides due to their age and feeding status [62, 63]. This scenario could also expose human to infective bites and possible malaria risk thus promoting residual malaria transmission under high intervention as earlier described [4]. Due to logistical reasons, circum-sporozoite detection could not be undertaken.

The predominant vector species identified was *An. coluzzii*, which is known to be highly endophilic and anthropophilic [64], however, the results here suggested that this vector population were displaying high zoophilic behaviour. The abundant presence of animals in the study areas and reduced access to human host due to intervention may have driven zoophagy and exophagy in this vector species as previously suggested [65, 66]. Further studies could explore the dynamics of this behaviour and its implication on the control efforts in the study region.

## Conclusions

This study demonstrated that *An. coluzzii* phenotypically resistant to DDT had a higher propensity for indoor-resting behaviour, while outdoor-resting tendency was found in those phenotypically-resistant to deltamethrin. Also, *An. coluzzii* with increased AChE and β-esterases activity, and *An. gambiae* s.s with *Vgsc*-*1014F* mutation displayed outdoor-resting behaviour. Mosquitoes resting indoors were found to have fed more on both human and animals than their outdoor counterparts. These findings highlight variation in response of mosquitoes within the same species to insecticide-based interventions. Continued monitoring of vector behaviours in surveillance programmes is recommended, to help in the control of malaria.

## Supplementary information

**Additional file 1: Table S1.** Number of indoor and outdoor test mosquitoes exposed and susceptibility rate to individual insecticides. Data on the number of mosquito populations exposed to insecticides and the susceptibility rate.

## Data Availability

All relevant data are within the paper. No supporting Information is available.

## Abbreviations

Ace-1: Acetylcholinesterase 1
DDT: Dichlorodiphenyltrichloroethane
DNA: Deoxyribonucleic acid
*GSTe2*: Glutathione-*s*-transferase epsilon 2
HBI: Human blood index
IRS: Indoor residual spraying
Kdr: Knockdown resistance
LLIN: Long-lasting insecticidal net
NMCP: National Malaria Control Programme
PCR: Polymerase chain reaction
PMI: President Malaria Initiative
SNP: Single nucleotide polymorphism
Vgsc: Voltage-gated sodium channel
WHO: World Health Organization
